# The Impact of Advertising Appeal Types on Purchase Intentions for Brands in Different Maturity Levels

**DOI:** 10.3390/bs16101926

**Published:** 2026-10-17

**Authors:** Xinliang Xu, Nannan Ji, Peipei Liu, Yixin Kang, Lin Yao, Shuxian Lai, Xianyou He, Wei Zhang

**Affiliations:** 1 Center for Studies of Psychological Application, South China Normal University, Guangzhou 510631, China; 2 Key Laboratory of Brain, Cognition and Education Sciences ( South China Normal University), Ministry of Education, Guangzhou 510631, China; 3 School of Psychology, South China Normal University, Guangzhou 510631, China; 4 Guangdong Key Laboratory of Mental Health and Cognitive Science, South China Normal University, Guangzhou 510631, China; 5 Philosophy and Social Science Laboratory of Reading and Development in Children and Adolescents, South China Normal University, Ministry of Education, Guangzhou 510631, China; 6 Department of Early Childhood Education, The Education University of Hong Kong, Hong Kong SAR, China

**Keywords:** advertising appeal, brand maturity, matching effect, purchase intention

## Abstract

Advertising research examines how advertising can influence consumer responses and purchase intentions. Drawing on the Elaboration Likelihood Model, this research examined whether functional and lifestyle advertising appeals differ in effectiveness for new and mature brands. Across six studies, Studies 1a and 1b examined consumers’ associations and preferences using free association and forced-choice tasks. Studies 2a and 2b then examined whether this matching pattern affected purchase intention using fictitious brands. Study 2b showed that image–text advertisements featuring lifestyle appeals elicited stronger purchase intentions for mature brands. Studies 3a and 3b further tested this effect using real brands and found that functional appeals were more effective for new brands, whereas lifestyle appeals increased purchase intentions for mature brands. Overall, the findings reveal a partial matching pattern between advertising appeals and brand maturity. Theoretically, this study identifies brand maturity as a boundary condition of advertising appeal effectiveness. Practically, the findings suggest that tailoring advertising appeals to brands’ developmental stages may enhance advertising effectiveness.

## 1. Introduction

In the current business environment, advertising is not merely a tool for conveying product information, but also a crucial means of shaping brand image and stimulating purchase intention (Huang et al., 2024; Gong & Seo, 2025). As advertising media evolve, advertising format shapes consumers’ information-processing patterns and persuasive outcomes (Sharma, 2026). However, faced with a wide range of commodities and advertisements, how do consumers make choices? And how does advertising influence consumers’ purchase decisions?

Advertising appeal is a core component of advertising messages and a key factor influencing advertising effectiveness (Holbrook & Batra, 1987; Shukla et al., 2022). Previous studies on advertising appeals have mainly focused on consumer traits, product characteristics, and advertising copy (Hoang et al., 2023; Kim et al., 2019; Wu et al., 2026). In contrast, brand maturity, which reflects a brand’s development process, directly influences consumers’ trust and loyalty toward the brand.

However, existing studies often overlook brand maturity when exploring how to maximize advertising effectiveness and stimulate consumers’ purchase intention. From a brand life cycle perspective, the fit between advertising appeals and brand maturity may therefore be important for advertising effectiveness. This study therefore examines whether brand maturity and advertising appeal interactively influence consumers’ purchase intention. Specifically, it investigates two questions: whether functional appeals increase purchase intentions more strongly for new brands and whether lifestyle appeals do so for mature brands.

## 2. Theoretical Background

### 2.1. Advertising Appeal Types

Advertising appeal is a key determinant of advertising effectiveness, influencing consumers’ cognition, emotions, and behaviors, which plays a significant role in the success of marketing (Teichert et al., 2018; X. Wang et al., 2025). Appeals are commonly categorized into functional appeals and lifestyle appeals (Massara et al., 2020). Functional appeals emphasize product attributes, performance, and practical benefits, thereby primarily eliciting cognitive responses (Kulkarni et al., 2020; J. Lee & Hong, 2016). Lifestyle appeals emphasize emotional experiences, imagination, and resonance with consumers’ lifestyles, eliciting affective responses and enabling the brand to serve as a means of self-expression (Keel & Nataraajan, 2012; O’ Hagan, 2022). Liu and Lei (2025) reported that virtual endorsers were more effective with rational appeals, whereas human endorsers were more effective with emotional appeals, with brand trust partially mediating endorsement effects on advertising effectiveness. Although rational-emotional and functional-lifestyle classifications are not identical, these findings illustrate why appeal effectiveness should be examined in relation to endorser characteristics and the advertising context.

According to the Elaboration Likelihood Model (ELM) proposed by Petty and Cacioppo (1986) and Ernst and Heesacker (1993), different advertising appeals exert their influence primarily through two distinct persuasive routes: the central route and the peripheral route. The central route involves deep cognitive processing, where consumers carefully evaluate message-relevant information, draw on prior knowledge, and integrate multiple sources to form purchase intentions. By contrast, the peripheral route involves lower cognitive effort, with consumers relying on non-content cues such as music, imagery, or endorser attractiveness to form attitudes rather than engaging with the actual arguments presented. Accordingly, the present research uses the ELM to motivate a possible information-processing account of appeal effectiveness.

Peripheral cues may include the number of message arguments, source characteristics, background music, and emotional responses evoked by the advertisement (M. T. Lee & Theokary, 2021; Shahab et al., 2021). When consumers choose the peripheral route to process advertising information, they do not engage in rigorous logical reasoning but instead rely more on peripheral cues in the advertisement to make decisions. From this theoretical perspective, functional advertisements may encourage central route processing, whereas lifestyle advertisements may encourage peripheral route processing by shaping brand image and lifestyle appealing to consumers’ lifestyles (Segev & Fernandes, 2023).

The two appeal types also differ significantly in linguistic style: functional advertisements tend to use concrete language to describe functions, whereas lifestyle advertisements predominantly employ abstract language to depict experiences (Massara et al., 2020). According to Construal Level Theory, these linguistic differences may prompt different construal levels when consumers interpret advertisements (Trope & Liberman, 2010). High-level construal emphasizes generalization and abstraction, while low-level construal emphasizes specific details and contexts (Kim et al., 2019; Trope & Liberman, 2003). Lifestyle advertisements promote higher construal levels by emphasizing abstract benefits, whereas functional advertisements promote lower construal levels by emphasizing concrete product attributes (Massara et al., 2020).

### 2.2. Brand Maturity

Brand maturity is a core concept in advertising that describes the stage of a product or service in the market life cycle (Hasaan et al., 2021). Kotler and Keller (2016) proposed that brands typically progress through introduction, growth, maturity, and potential decline. Consumers perceive brand maturity through direct and indirect experiences that shape brand familiarity, memory, and associations (Davtyan et al., 2021; de Lencastre et al., 2023; Schmidt & Eisend, 2015). Brand maturity is distinct from brand familiarity, which refers to the extent of consumers’ direct and indirect experiences with a given brand (Rosengren et al., 2020). Although the two may covary, they are not interchangeable. Through emotional contagion, consumers’ feelings toward things associated with a familiar brand may transfer to that brand. For example, positive feelings toward a sporting event may transfer to its sponsoring brand. Consumers may maintain favorable brand attitudes when a familiar brand can consistently meet their needs with high-quality products and services, or when its advertising aligns with their expectations; otherwise, they may develop unfavorable attitudes (Boronczyk & Breuer, 2020; Garczarek-Bąk et al., 2021). For unfamiliar brands, the lack of experience inhibits consumers’ information processing and attitude formation (Davtyan et al., 2021). Firms therefore need marketing strategies to adapt to each developmental stage (Pauwels et al., 2021). Overall, Brand maturity reflects a brand’s market longevity, accumulated market presence and developmental trajectory (Ladeira et al., 2022).

In this study, we focus on the introduction and maturity stages of the brand lifecycle, corresponding to new and mature brands, respectively. New brands typically have limited brand awareness and fewer established associations, making it important to communicate product-related information that helps consumers understand the brand and its offerings. Accordingly, marketing efforts at this stage often emphasize product differentiation, functional benefits, and clear positioning. In contrast, during the maturity stage, brands have accumulated awareness and a stable customer base; thus, marketing shifts toward strengthening brand image, reputation, and consumer loyalty by fostering deeper connections with consumers.

Prior research also suggests that advertising appeals should be considered in relation to market or brand maturity. Chandy et al. (2001) examined the role of advertising appeals in emerging and mature markets and proposed that advertising strategies should be aligned with the maturity of the market to enhance advertising effectiveness. When consumers evaluate products from new brands, they prioritize the functional attributes before experiential value.

### 2.3. Purchase Intention

Purchase intention refers to consumers’ subjective inclination to choose a specific product and reflects their preference during the decision-making process. It has been shown to be an important predictor of actual purchase behavior because it reflects consumers’ interest in and possibility of purchasing a product. Purchase intention also helps explain why consumers choose a specific brand (Ahmad & Dubey, 2026; Peña-García et al., 2020; Shah et al., 2012).

Purchase decision involves consumers’ cognition, attitudes, and behaviors. As an antecedent of actual purchase behavior, purchase intention is widely used to predict consumers’ decisions, although it may be influenced by consumer identity, price, perceived quality, and value (Casteran, 2026; Fang et al., 2026). Brand-related factors, including brand image and awareness, also shape purchase intention (Azzari & Pelissari, 2020; Uppapong et al., 2026; Usman & Permatasari, 2019).

In addition, advertising allows consumers to learn about a brand’s products, and appropriate advertising can improve promotional effectiveness and boost purchase intention. Brand familiarity affects how consumers process advertising: existing product knowledge can facilitate information processing. However, familiarity may also encourage peripheral route processing and reduce elaboration of information content and rigorous reasoning (Petty & Wegener, 1998).

## 3. Development of Hypotheses

Prior research suggests that the effectiveness of advertising appeals depends partly on consumers’ prior knowledge (Aikman et al., 2006; Olson & Zanna, 1993). Consumers with little product knowledge actively process advertising arguments to reduce uncertainty and risk, making functional appeals more persuasive via concrete factual cues. Lifestyle emotional appeals are less effective in this context because they provide less diagnostic information about unfamiliar products. For consumers with greater knowledge and pre-existing attitudes, the need for extensive information processing diminishes; lifestyle appeals then dominate by grabbing attention and activating stored brand perceptions to drive purchase intentions.

**Figure 1:**
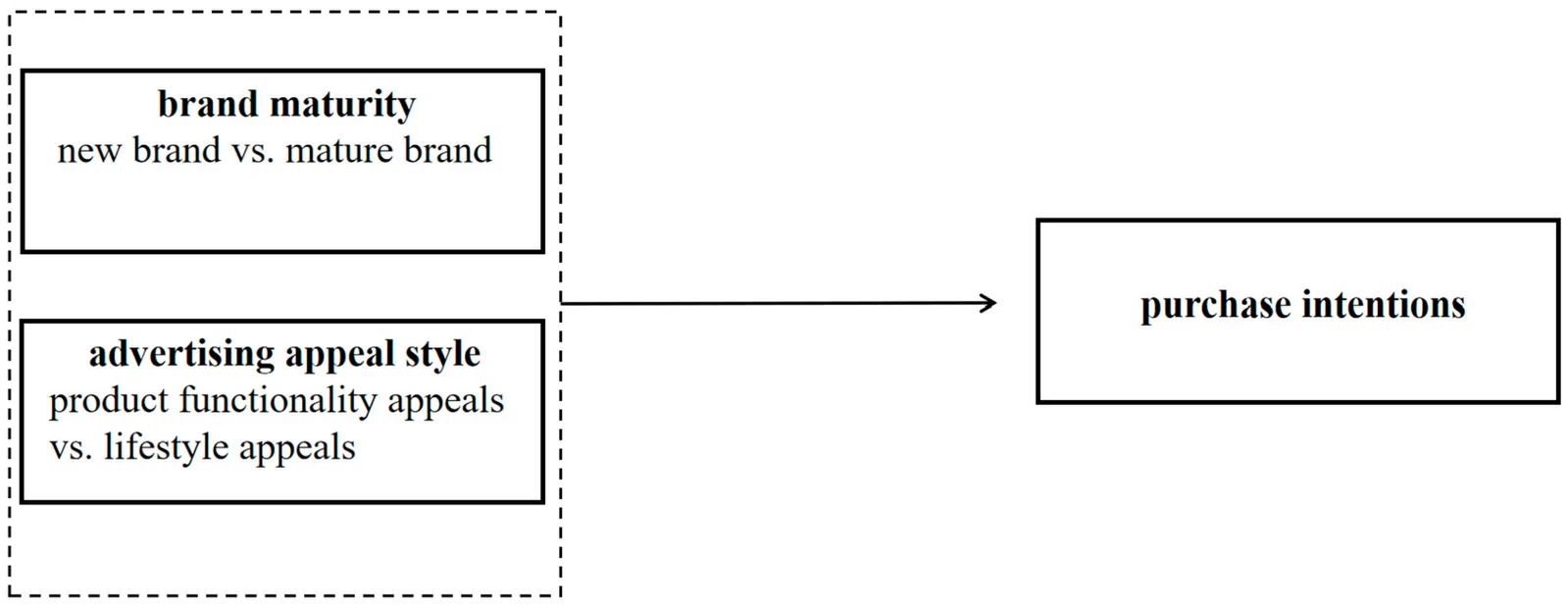
Conceptual framework of this study.

Supporting evidence exists in research on brand familiarity: when advertisements focus on lifestyle appeals, consumers evaluate familiar brands more positively; in contrast, when advertisements emphasize functional appeals, consumers tend to evaluate products from unfamiliar brands relatively more favorably (Rhee & Jung, 2019). B. Yang et al. (2021) found that rational appeals for a healthy food item were more attractive under low uncertainty, whereas no significant difference between rational and emotional appeals emerged under high uncertainty (Figure 1).

Based on the aforementioned theoretical principles and empirical evidence, we propose the following hypotheses:

**H1.**  *For new brands, functional advertising appeals are more effective at enhancing consumers’ purchase intention. In contrast, lifestyle advertising appeals yield higher purchase intention for mature brands.*

**H2.**  *The matching effect between advertising appeal type and brand maturity will be consistent across both textual advertising slogans and image–text poster advertisements.*

## 4. Overview of Studies

**Figure 2:**
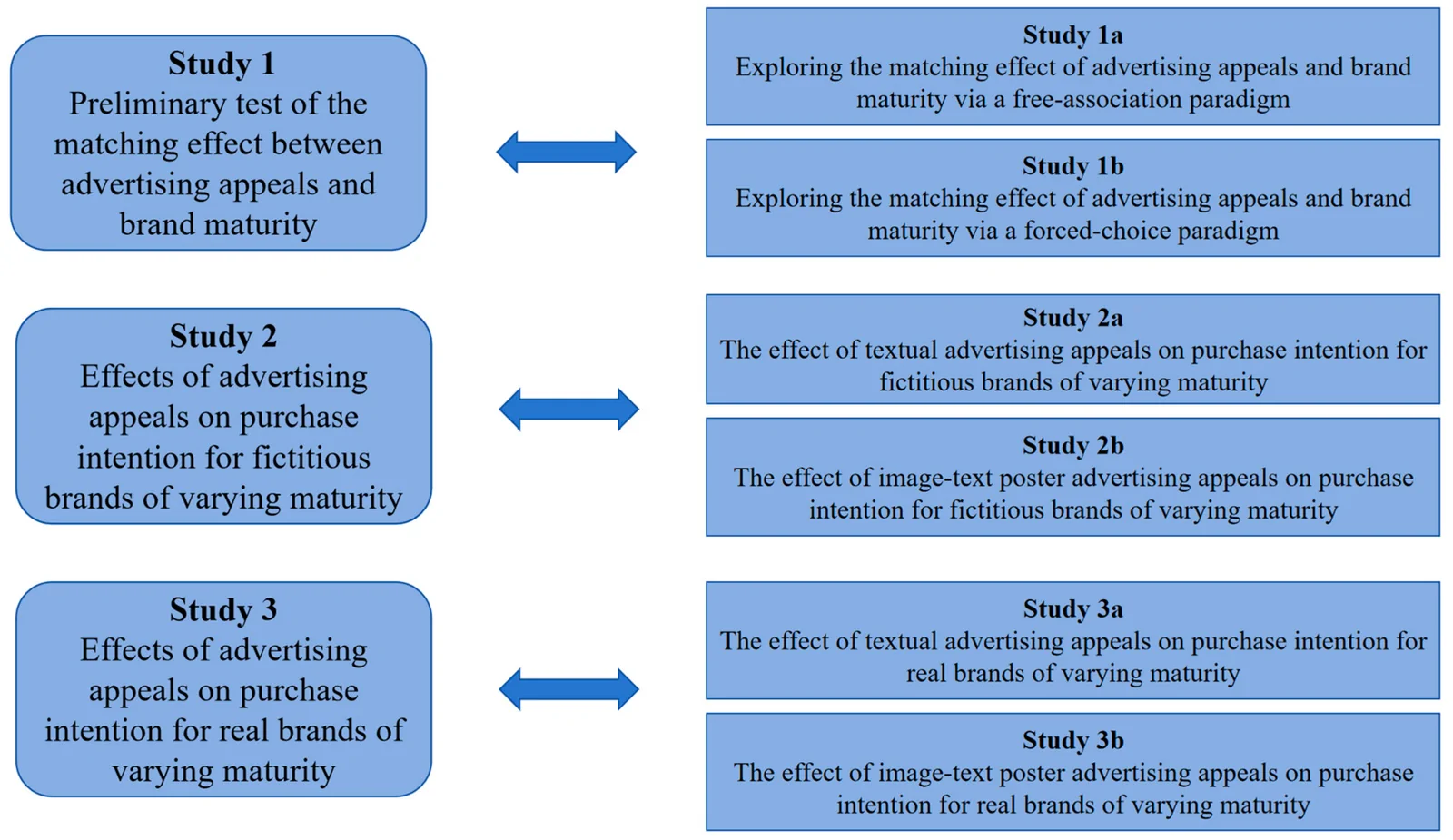
Research framework of this study.

This research includes three studies covering six experiments, designed to examine how advertising appeal types affect purchase intention across brands of different maturity levels and further develop tailored advertising strategies for brands in distinct life cycles. Studies 1a and 1b adopted free association and forced-choice tasks to preliminarily explore whether consumers hold differential preferences for advertising appeals of brands with varying maturity. Studies 2a and 2b employ fictitious brands to achieve examination of the core matching effect. These two sub-experiments test the influence of ad appeal types on purchase intention by adopting text-only slogans and image–text posters, respectively. Building on these findings, Studies 3a and 3b further utilize real market brands to improve the ecological validity of the research and verify the stability of the observed matching effect. Consistent with Study 2, both textual slogans and image–text posters are adopted in Study 3 to compare and analyze how different advertising appeals shape consumers’ purchase intention for new versus mature brands. The parallel design of two distinct advertising formats across Studies 2 and 3 serves to test the robustness of the core effect, ensuring that the research conclusions are generalizable and not restricted to a single advertising presentation form (Figure 2).

## 5. Study 1a

Study 1a employed a free association paradigm to examine how advertising appeals are associated with brands of different maturity. We assume that participants would associate new brands more with functional attributes, and mature brands more with lifestyle and emotional attributes.

### 5.1. Participants and Procedure

A total of 40 participants (*M*_age_ = 20.55, *SD* = 2.16; 31 females) were recruited online. Participants were screened using the following criteria: non-psychology majors, normal or corrected-to-normal vision, and no history of mental illness. All participants took part voluntarily and received compensation upon completing the experiment. All experiments in this study recruited participants following the above criteria. To avoid carry-over effects, every participant in this study joined only one experiment. Participants were each randomly assigned to one of two between-subjects conditions of brand maturity (new brand vs. mature brand).

Study 1a was an online experiment. Participants were presented with a set of advertising-appeal keywords covering six product categories: food, beauty and personal care, beverages, dairy products, electronics, and uncategorized products. The keyword set contained 36 food-related keywords, 34 beauty and personal care keywords, 34 beverage-related keywords, 30 dairy-related keywords, 22 electronics-related keywords, and 34 uncategorized keywords. Half of the keywords described functional attributes, whereas the other half described lifestyle-related attributes. The participants were asked to select five advertising keywords for each category for new brands and mature brands, according to their own feelings and impressions. Based on the selected keywords, word clouds were generated using Word It Out.

### 5.2. Results and Discussion

**Figure 3:**
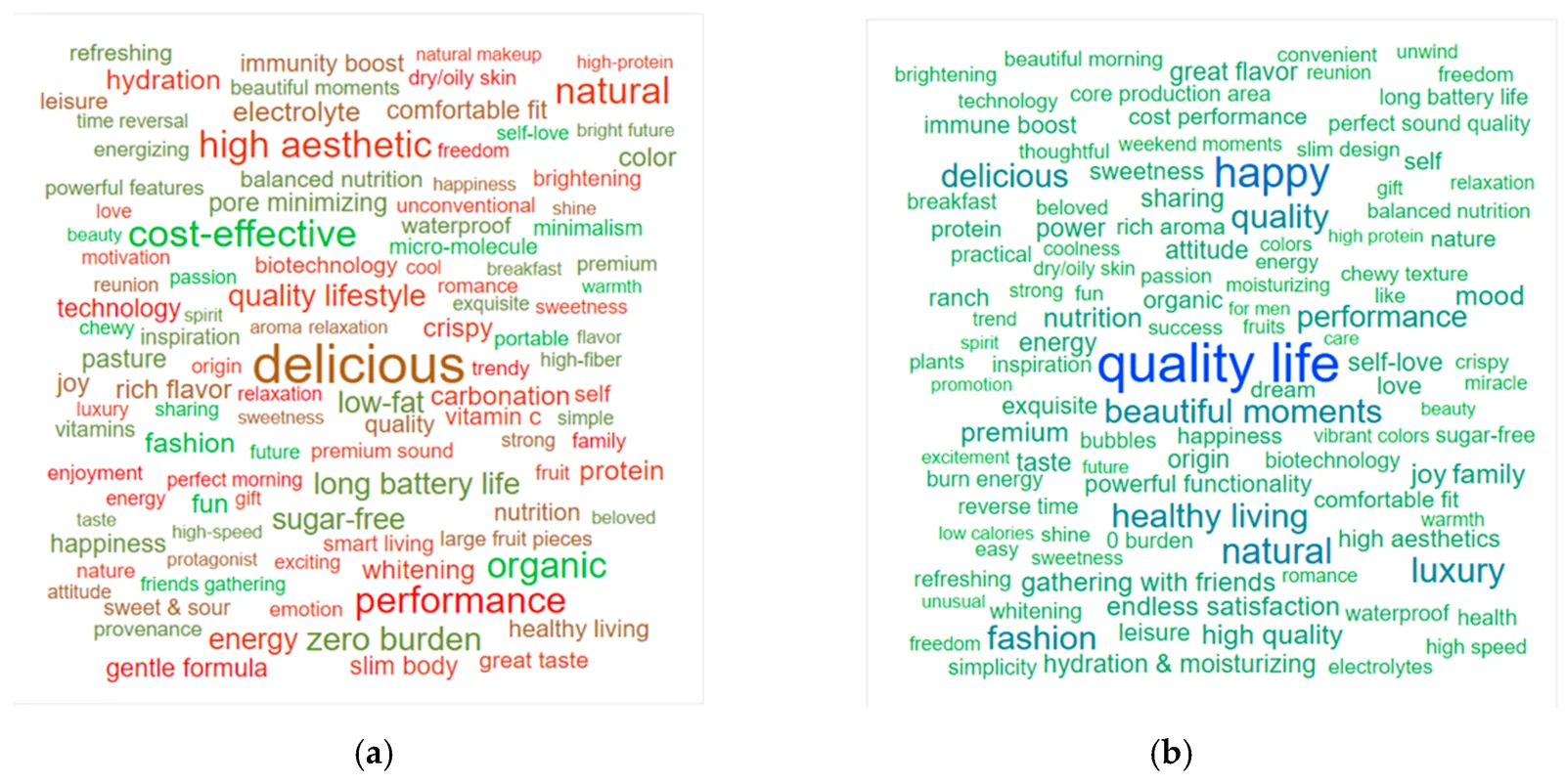
Word Clouds of the new brand (**a**) and mature brand (**b**).

The selected advertising keywords of new brands and mature brands were put into Word It Out respectively to generate the word cloud map (see Figure 3). In the word cloud map of the new brand, the five most frequently selected words are “delicious”, “cost-effective”, “performance”, “high aesthetic “ and “natural”; In the word cloud map of mature brands, the five most frequently selected words are “quality life”, “happy”, “natural”, “luxury” and “fashion”.

The results of study 1a showed that people have different impressions of the advertisements of new brands and mature brands. People are more likely to associate the advertisements of new brands with keywords that emphasize the functions and performance of products. In addition, they are also more likely to associate the advertisements of mature brands with keywords that emphasize lifestyle, emotion, and attitude. In order to further verify this selection tendency, we will adopt the forced selection method in Study 1b to verify people’s preference for advertising appeal types of brands with different maturity.

## 6. Study 1b

Study 1b aimed to explore people’s interest in advertising appeal types of new brands and mature brands in a forced selection task. We assume that if it is a new brand, people may have less understanding of the brand and its products, so people will be more interested in functional advertisements and will be more likely to watch functional advertisement posters. If it is a mature brand, people have a good understanding of the brand and the product, so they will be more likely to watch the product-based lifestyle advertising posters.

### 6.1. Participants and Procedure

Thirty-seven college students (*M*_age_ = 21.16, *SD* = 2.27) were recruited. Five participants were excluded for failing the attention check, leaving 32 participants (16 females) for the subsequent analysis.

Study 1b adopted a 2 (brand maturity: new brand vs. mature brand) × 2 (advertising appeal: functional appeal vs. lifestyle appeal) within-subjects design, and the dependent variable was the number of participants’ choices.

Real-market brands were selected according to predefined criteria. New brands had been launched within the previous three years and had relatively low public awareness, whereas mature brands had more than 20 years of market history, an established reputation, and widespread recognition. Thirty-four participants (25 males, 9 females; *M*_age_ = 25.44, *SD* = 2.68) rated candidate brands on two seven-point items measuring perceived newness and maturity. Twenty new and twenty mature brands were retained based on ratings. Paired-samples *t* tests confirmed the validity of the brand selection. New brands received significantly higher newness ratings (*M* = 4.35, *SD* = 1.51) than mature brands (*M* = 2.58, *SD* = 1.27), *t*(33) = 4.82, *p* < 0.001. Meanwhile, mature brands received significantly higher maturity ratings (*M* = 6.20, *SD* = 0.51) than new brands (*M* = 2.52, *SD* = 1.06), *t*(33) = −18.26, *p* < 0.001.

We also created brand descriptions for new and mature brands according to their brand and product introductions. Examples of the descriptions are listed as follows. New brand introduction: “Little Prince” is a potato chip brand, which is a new brand launched in the market in the past three years. The main product of this brand is non-fried potato chips; the target group is young people. The product’s selling points are taste, crispiness, and health. Mature brand introduction: “Copic” is a potato chip brand which has more than 20 years of mature brand history. The main product of this brand is non-fried potato chips; the target group is young people. The product’s selling points are taste, crispiness, and health.

Study 1b was programmed using E-Prime 3.0. All stimuli were presented at the center of the screen on a black background (1024 × 768 pixels resolution). Participants were asked to pay attention to a 600 ms fixation point “+” at the center of the screen. A total of 40 trials were randomly presented to the participants.

On each trial, participants read self-paced brand introductions containing brand name, maturity information, and product selling points. The corresponding new and mature brands were matched on product selling points. After reading each description, participants completed a brand-maturity manipulation check to assess whether they had correctly understood the brand’s maturity level. Participants then reviewed definitions of functional and lifestyle advertising appeals and selected their preferred poster type. The presentation order of the two appeal types was randomized to counteract order effects.

### 6.2. Results and Discussion

Chi-square analysis conducted via SPSS 26.0 revealed significant differences in ad appeal choices across brand maturity levels, χ^2^ (1) = 15.65, *p* < 0.001. Pairwise comparisons showed that participants selected functional appeals more frequently than lifestyle appeals for new brands, with 191 selections (59.69%) for functional appeals and 129 selections (40.31%) for lifestyle appeals, *p* < 0.05. Conversely, lifestyle advertisements were selected more frequently for mature brands (179 selections, 55.94%) than functional advertisements (141 selections, 44.06%), *p* < 0.05.

Study 1b preliminarily indicated that people have obvious preferences for advertising appeal styles of brands with different maturity, that is, participants are more interested in new brands with functional advertisements and mature brands with lifestyle advertisements. Advertising slogans are widely used in brand communication, and pictures and text are key elements of print advertisements (Pieters & Wedel, 2004; Kohli et al., 2007). Study 2a used textual advertising slogans, whereas Study 2b used image–text advertisements. Both studies used fictitious brands to examine the effect of advertising appeal type on purchase intention across new and mature brand conditions.

## 7. Study 2a

In Study 2a, we tried to use textual advertisements as materials to explore the influence of textual slogans with different advertising appeals on the purchase intention of different mature brands. The hypothesis of Study 2a is similar to Study 1b. We predicted that textual advertisements with product function can significantly enhance people’s purchase intention for new brands. However, we predicted that lifestyle textual advertisements will significantly facilitate their purchase intention for mature brands.

### 7.1. Participants and Procedure

Based on G*Power 3.1 calculations, for the within-subjects repeated-measures ANOVA used in this study, a minimum total sample size of 36 participants was projected to achieve 95% statistical power (1 − β) at α = 0.05 with a medium effect size of *f* = 0.25. A total of 60 participants (*M*_age_ = 21.36, *SD* = 2.50) were recruited online in this study. Five participants were excluded because they failed the attention check, leaving 55 participants (29 females) for the subsequent analysis.

For the rating of textual advertising slogans, 24 participants who did not take part in the formal study were recruited. They rated the extent to which each textual slogan emphasized product function (7-point scale: 1 = more likely to highlight lifestyle, 7 = more likely to highlight product function) in response to the question, “Do you think this textual slogan expresses the product’s lifestyle or function?” Based on the rating results, 44 textual slogans (22 functional and 22 lifestyle slogans) were selected. The paired sample *t*-test showed that the functional slogans received significantly higher functional-attribute ratings (*M* = 5.82, *SD* = 0.67) than the lifestyle slogans (*M* = 3.35, *SD* = 0.77), *t*(23) = 11.72, *p* < 0.001. All the materials were divided into four sets of stimuli. Each set of stimuli had 11 advertising slogans. Examples of the four sets of stimuli were as follows.

Functional advertisement slogan for a new brand:

The following is the advertising slogan of a yogurt brand launched in 2023. The main product is a healthy and delicious fruit yogurt; the target group is young people; the selling points of the product are its fragrance and healthiness; the brand is positioned as young and energetic. The slogan is: “Full of oversized fruit!”

Lifestyle advertisement slogan for a new brand:

The following is the advertising slogan of a yogurt brand launched in 2023. Its main product is a healthy and delicious fruit yogurt; its main target group is young people; the selling points of the product are its fragrance and healthiness; the brand is positioned as young and energetic. The slogan is: “Young of its own school!”

Functional advertisement slogan for a mature brand:

The following is the advertising slogan of a yogurt brand with a 20-year history and a certain level of brand awareness. The main product is a healthy and delicious fruit yogurt; the target group is young people; the selling points of the product are its fragrance and healthiness; the brand is positioned as young and energetic. The slogan is: “Chew to get the great satisfaction of fruit!”

Lifestyle advertisement slogan for a mature brand:

The following is the advertising slogan of a yogurt brand with 20 years of history and a certain level of brand awareness. The main product is a healthy and delicious fruit yogurt; the target group is young people; its selling points are its fragrance and healthiness; the brand is positioned as young and energetic. The slogan is: “Borderless taste tour!”

To measure participants’ willingness to purchase the advertised products, the present study adopted the Consumer Purchase Intention Scale (Aaker et al., 2010), as translated into Chinese by C. Yang (2018). Using a 7-point scale (1 = strongly disagree, 7 = strongly agree), α = 0.847, and it originally included three items: (1) “I am interested in buying the advertised product”; (2) “On the whole, I am very satisfied with the product in the advertisement”; (3) “I am very likely to buy the advertised product”. While all three items belong to the original Consumer Purchase Intention Scale, they capture different facets of the broader construct. Items 1 and 3 directly assess behavioral purchase intentions, which is the focal construct of our research. By contrast, Item 2 taps attitudinal evaluation of the advertised product. To focus strictly on behavioral purchase intentions, we retained Items 1 and 3 for analyses. The Cronbach’s α of this two-item scale in Study 2a and subsequent studies was above 0.8.

Study 2a employed an online within-subjects experiment with a 2 (brand maturity: new vs. mature) × 2 (advertising appeals: functional vs. lifestyle) design. Participants completed 44 randomly ordered trials following a procedure consistent with Study 1b. After completing the manipulation check, each brand slogan was displayed, and participants rated their purchase intention.

### 7.2. Result and Discussion

#### 7.2.1. Purchase Interest

For purchase interest, the main effect of brand maturity was marginally significant, *F*(1, 54) = 4.03, *p* = 0.050, η^2^ = 0.07. Purchase interest was somewhat higher for the mature brand (*M* = 4.70, *SD* = 0.80) than for the new brand (*M* = 4.62, *SD* = 0.85). The main effect of advertising appeal was also significant, *F*(1, 54) = 5.29, *p* = 0.025, η^2^ = 0.09. Purchase intention was higher for the functional slogan (*M* = 4.75, *SD* = 0.97) than for the lifestyle slogan (*M* = 4.57, *SD* = 0.82). However, the interaction between brand maturity and advertising appeal type was not significant, *F*(1, 54) = 0.03, *p* = 0.868, η^2^ < 0.01.

#### 7.2.2. Purchase Possibility

For purchase possibility, there was a significant main effect of brand maturity, *F*(1, 54) = 7.69, *p* = 0.008, η^2^ = 0.13. Purchase intention was higher for mature brands (*M* = 4.60, *SD* = 0.08) than for new brands (*M* = 4.47, *SD* = 0.09). A significant main effect of advertising appeal also emerged, *F*(1, 54) = 12.01, *p* = 0.001, η^2^ = 0.18. Purchase intention was higher for functional slogans (*M* = 4.66, *SD* = 0.10) than for lifestyle slogans (*M* = 4.41, *SD* = 0.09). However, the interaction between brand maturity and advertising appeal was not significant, *F*(1, 54) = 0.04, *p* = 0.840, η^2^ < 0.01.

These findings did not support our hypothesis, as no interaction between brand maturity and advertising appeal was observed. We speculated that text-only slogans may provide relatively limited contextual and visual information, leading participants to rely more heavily on concrete product-related information. To test this conjecture, Study 2b used image–text advertisements to further examine the effect of advertising appeal on purchase intention across different levels of brand maturity.

## 8. Study 2b

Study 2b used image–text advertisements to further examine whether the matching effect between advertising appeal and brand maturity would emerge when advertisements provided both visual and textual information. The hypotheses of Study 2b were the same as those of Study 2a.

### 8.1. Participants and Procedure

Based on G*Power 3.1 calculations, for the mixed-design repeated-measures ANOVA used in this study, a minimum total sample size of 54 participants was projected to achieve 95% statistical power (1 − β) at α = 0.05 with a medium effect size of *f* = 0.25. A total of 118 participants (*M*_age_ = 20.70, *SD* = 2.16) were recruited for Study 2b. Eight were excluded for failing the attention check, leaving 110 participants (65 females) for data analysis.

The image–text advertisements were developed using the slogans from Study 2a. These posters were designed by a professional advertising designer based on the brand and slogan materials from Study 2a. To minimize stylistic variation across stimuli, all posters were developed by the same designer.

**Figure 4:**
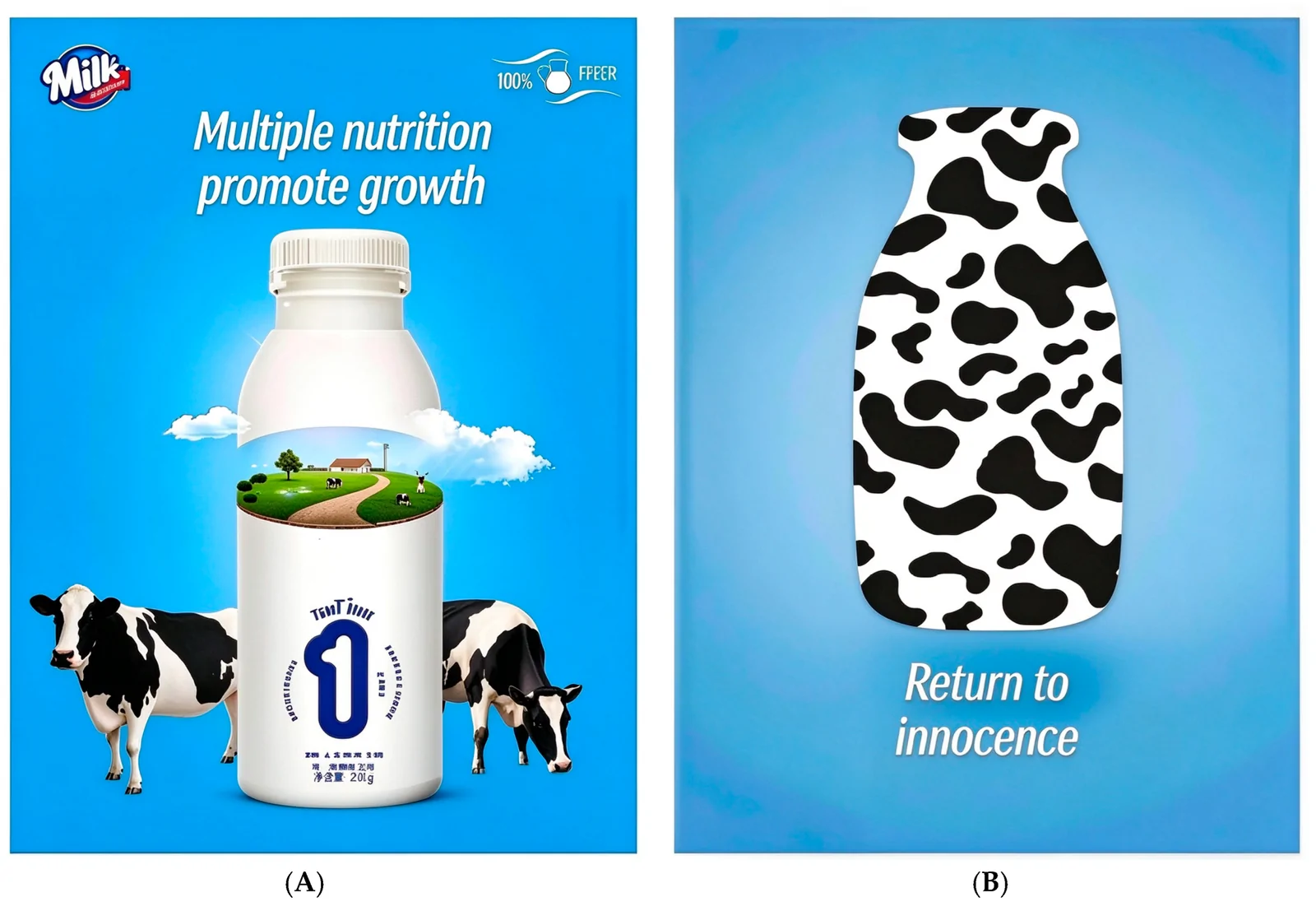
Examples of functional advertisement poster (**A**) and lifestyle advertisement poster (**B**).

Thirty participants (13 males) who did not take part in the main study rated the image–text advertisements using the same procedure as in Study 2a. Based on these ratings, 40 posters were selected, including 20 functional and 20 lifestyle advertisements. A paired-samples *t*-test showed that perceived functional attributes were significantly higher in the functional condition (*M* = 5.74, *SD* = 0.58) than in the lifestyle condition (*M* = 3.36, *SD* = 1.07), *t*(29) = 9.29, *p* < 0.001. Examples of brand descriptions are provided below, and sample posters are shown in Figure 4.

New brand introduction:

The brand is a milk brand, which is a new brand to be launched in 2023. The main product is milk; the target group is families; the product selling point is natural pasture and high quality; the brand spirit is quality and natural!

Mature brand introduction:

The brand is a milk brand and is a mature brand with 20 years of brand history and has a certain brand awareness. The main product is milk; The target group is families; the product selling point is natural pasture and high quality; the brand spirit is quality and natural!

To eliminate poster familiarity bias, we adopted a mixed design: advertising appeal type served as the within-subjects factor, while brand maturity acted as the between-subjects factor.

**Figure 5:**
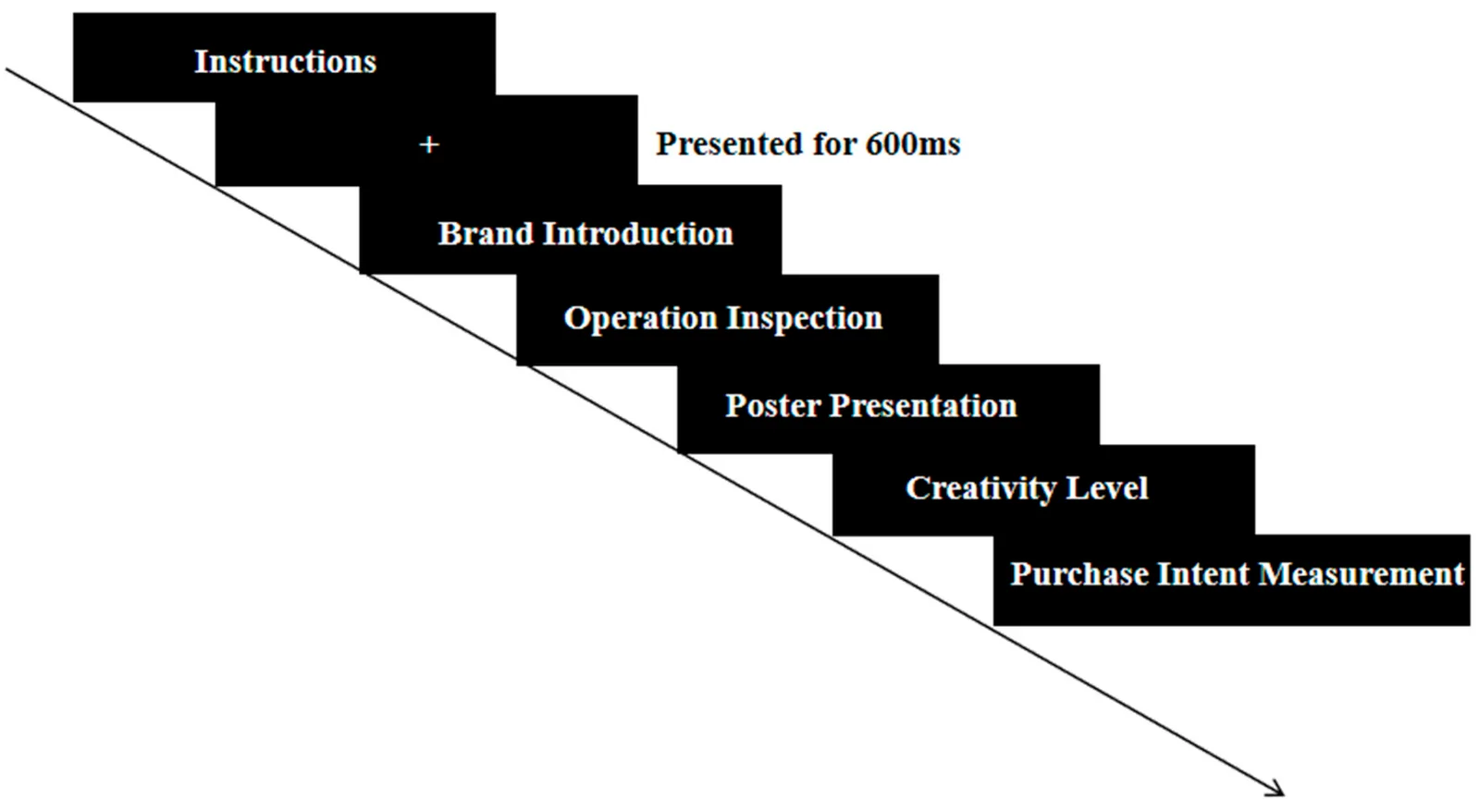
Schematic diagram of the procedure for Study 2b.

The experiment was programmed with E-Prime 3.0. Stimuli appeared centrally on a 541 × 768 black-background screen. Each trial started with a 600 ms fixation cross, followed by brand descriptions and a brand maturity manipulation check. Participants then viewed the image–text advertisement at their own pace and rated its creativity and their purchase intention using seven-point scales consistent with those used in Study 2a. Stimulus sequences were fully randomized to mitigate order effects (see Figure 5).

### 8.2. Results and Discussion

#### 8.2.1. Purchase Interest

For purchase interest, the main effect of advertising appeal type was significant, *F* (1, 108) = 5.35, *p* = 0.023, η^2^ = 0.05, whereas brand maturity yielded no significant main effect, *F*(1, 108) = 0.20, *p* = 0.653, η^2^ < 0.01. The interaction between brand maturity and advertising appeal was significant, *F*(1, 108) = 4.15, *p* = 0.044, η^2^ = 0.04.

Simple effect analyses supported our hypothesis: lifestyle posters generated greater purchase interest for mature brands (*M* = 4.40, *SD* = 0.85) than functional posters (*M* = 4.16, *SD* = 0.85), *F*(1, 108) = 9.46, *p* = 0.003. No significant difference emerged for new brands between functional (*M* = 4.21, *SD* = 0.78) and lifestyle posters (*M* = 4.22, *SD* = 0.84), *F*(1, 108) = 0.04, *p* = 0.845.

**Figure 6:**
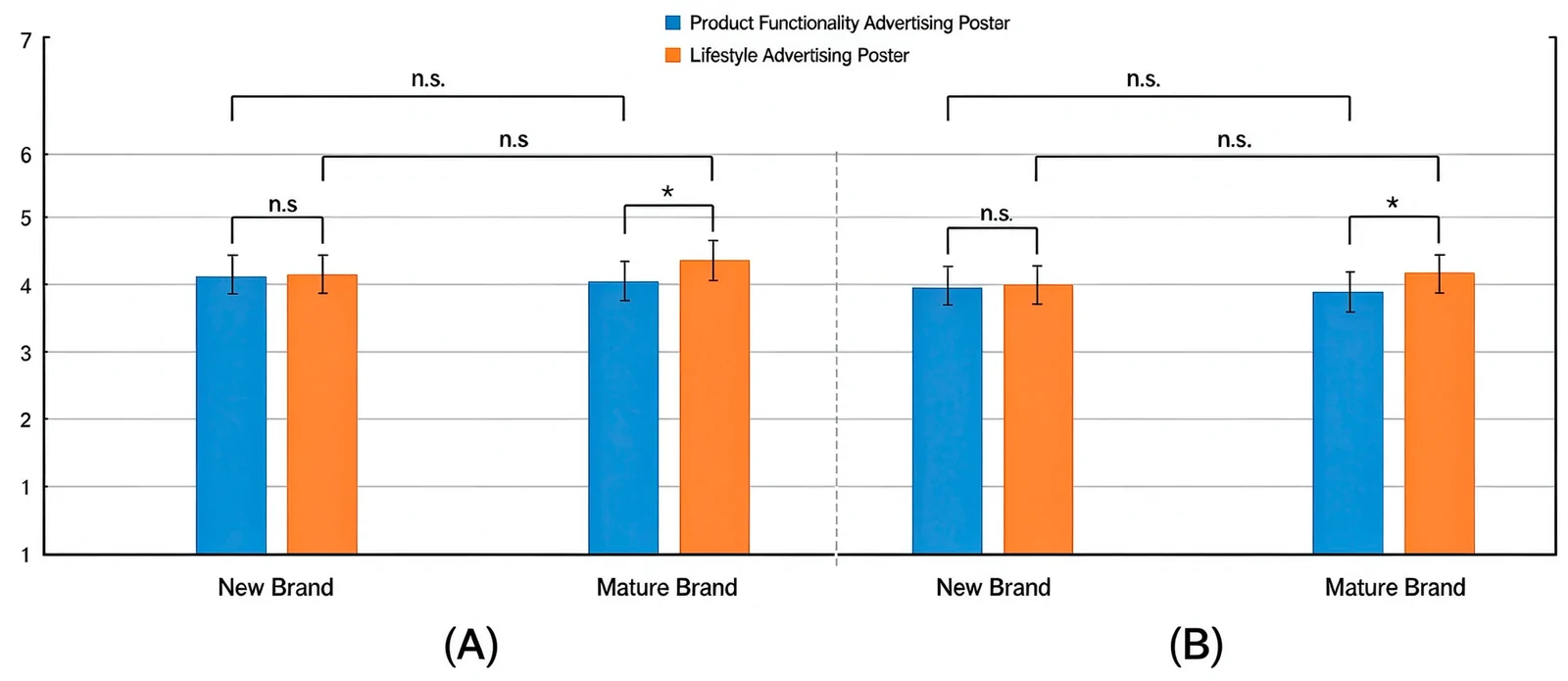
Purchase interest (**A**) and purchase possibility (**B**) under various experimental treatments of Study 2b. * represents *p* < 0.05, and n.s. represents non-significant.

Purchase intention did not differ between new and mature brands under either functional condition, *F*(1, 108) = 0.08, *p* = 0.785, or under the lifestyle poster condition, *F*(1, 108) = 1.21, *p* = 0.275 (see Figure 6).

#### 8.2.2. Purchase Possibility

For purchase possibility, advertising appeal type exerted a significant main effect, *F*(1, 108) = 4.13, *p* = 0.045, η^2^ = 0.04, while brand maturity had no significant main effect, *F*(1, 108) = 0.02, *p* = 0.884, η^2^ < 0.01. Their interaction was marginally significant, *F*(1, 108) = 3.80, *p* = 0.054, η^2^ = 0.03.

Simple effect tests showed that, among mature brands, purchase intention was higher for lifestyle advertising (*M* = 4.22, *SD* = 0.87) than for functional advertising (*M* = 4.00, *SD* = 0.86), *F*(1, 108) = 7.93, *p* = 0.006. However, there was no significant difference in purchase possibility of new brands between functional advertising (*M* = 4.10, *SD* = 0.73) and lifestyle advertising (*M* = 4.09, *SD* = 0.83), *F*(1, 108) < 0.01, *p* = 0.954.

In the functional advertising condition, there was no significant difference in purchase possibility between new brands and mature brands, *F*(1, 108) = 0.29, *p* = 0.590. In the lifestyle advertising condition, the difference in purchase possibility between new brands and mature brands was also not significant, *F* (1, 108) = 0.65, *p* = 0.421 (see Figure 6).

Unlike Study 2a, Study 2b supported our hypotheses: lifestyle image–text advertisements boosted mature brands’ purchase interest and possibility, yet appeal exerted no influence on new brands. We attributed this inconsistency to fictitious brands impairing maturity perception. Studies 3a and 3b therefore adopt real brands to retest advertising appeal on purchase intention across different levels of brand maturity.

## 9. Study 3a

Study 3a used real brand names as materials to explore the influence of textual advertising slogans with different advertising appeal types on the purchase intention of brands with different maturity. The hypotheses of Study 3a were identical to those of Study 2a.

### 9.1. Participants and Procedure

Based on G*Power 3.1 calculations, for the within-subjects repeated-measures ANOVA used in this study, a minimum total sample size of 36 participants was projected to achieve 95% statistical power (1 − β) at α = 0.05 with a medium effect size of *f* = 0.25. A total of 49 participants (*M*_age_ = 20.68, *SD* = 1.65) were recruited online. Four were excluded for failing the attention check, leaving 45 (23 females) for subsequent analysis.

Study 3a used the same experimental design as Study 2a and followed a similar procedure, but used real brand names as experimental materials.

Brand pre-rating followed the procedure of Study 1b, yielding 13 new and 13 mature real brands. Paired-samples *t*-test validated the manipulation: new brands received higher novelty ratings (4.22 ± 1.54) than mature brands (2.67 ± 1.45), *t*(33) = 4.57, *p* < 0.001; mature brands earned higher maturity ratings (6.07 ± 0.61) than new brands (2.53 ± 1.06), *t*(33) = −17.35, *p* < 0.001.

Text slogans tailored to these real brands were screened using the rating protocol of Study 2a, retaining 26 functional and 26 lifestyle slogans. Functional slogans scored significantly higher on functional emphasis (*M* = 5.90, *SD* = 0.65) than lifestyle ones (*M* = 3.24, *SD* = 0.82), *t*(23) = 12.11, *p* < 0.001. The slogans were matched to brand types with 13 items per condition. Examples are provided below.

Functional advertisement slogan for new brand:

“Little Prince” is a brand of potato chips. It is a new brand launched in the market within the past three years. The brand spirit is fun, young and energetic; the brand launched its main product, non-fried potato chips, aimed at young people; the selling points of the product are its taste, crispiness, and healthiness. The slogan is: “’Ka-ching’ crisp!”

Lifestyle advertisement slogan for new brand:

“Little Prince” is a potato chip brand, which is a new brand launched in the market in the past three years. The brand spirit is fun, young and dynamic; the brand launched its main product, non-fried potato chips, aimed at young people; the selling points of the product are its taste, crispiness, and healthiness. The slogan is: “Lots of fun!”

Functional advertisement slogan for a mature brand:

“Kopic” is a potato chip brand with more than 20 years of brand history as a mature brand; brand spirit: fun, young, dynamic; the brand launched its main product, non-fried potato chips, aimed at young people; the selling points of the product are its taste, crispiness, and healthiness. The slogan is: “Pure ‘crisp’ natural good taste!”

Lifestyle advertisement slogan for mature brand:

“Kopic” is a potato chip brand with more than 20 years of brand history, a mature brand, with a brand spirit for fun, young, and dynamic; the brand launched its main product, non-fried potato chips, aimed at young people; the selling points of the product are its taste, crispiness, and healthiness. The slogan is: “‘Potato” in my happy moment!’

### 9.2. Results and Discussion

#### 9.2.1. Purchase Interest

The results of purchase interest showed that the main effect of brand maturity was significant, *F*(1, 44) = 21.23, *p* < 0.001, η^2^ = 0.33. The main effect of advertising appeal type was not significant, *F*(1, 44) = 0.53, *p* = 0.473, η^2^ = 0.01. The interaction between brand maturity and advertising appeal types was significant, *F*(1, 44) = 57.13, *p* < 0.001, η^2^ = 0.56.

The simple effects analysis showed that the purchase interest of new brands elicited by the functional slogan (*M* = 4.73, *SD* = 0.87) was significantly higher than that elicited by the lifestyle slogan (*M* = 4.37, *SD* = 0.96), *F* (1, 44) = 11.18, *p* = 0.002. On the contrary, the purchase interest of mature brands elicited by the lifestyle slogan (*M* = 5.04, *SD* = 0.87) was significantly higher than that elicited by the functional slogan (*M* = 4.81, *SD* = 0.80), *F*(1, 44) = 7.11, *p* = 0.011.

**Figure 7:**
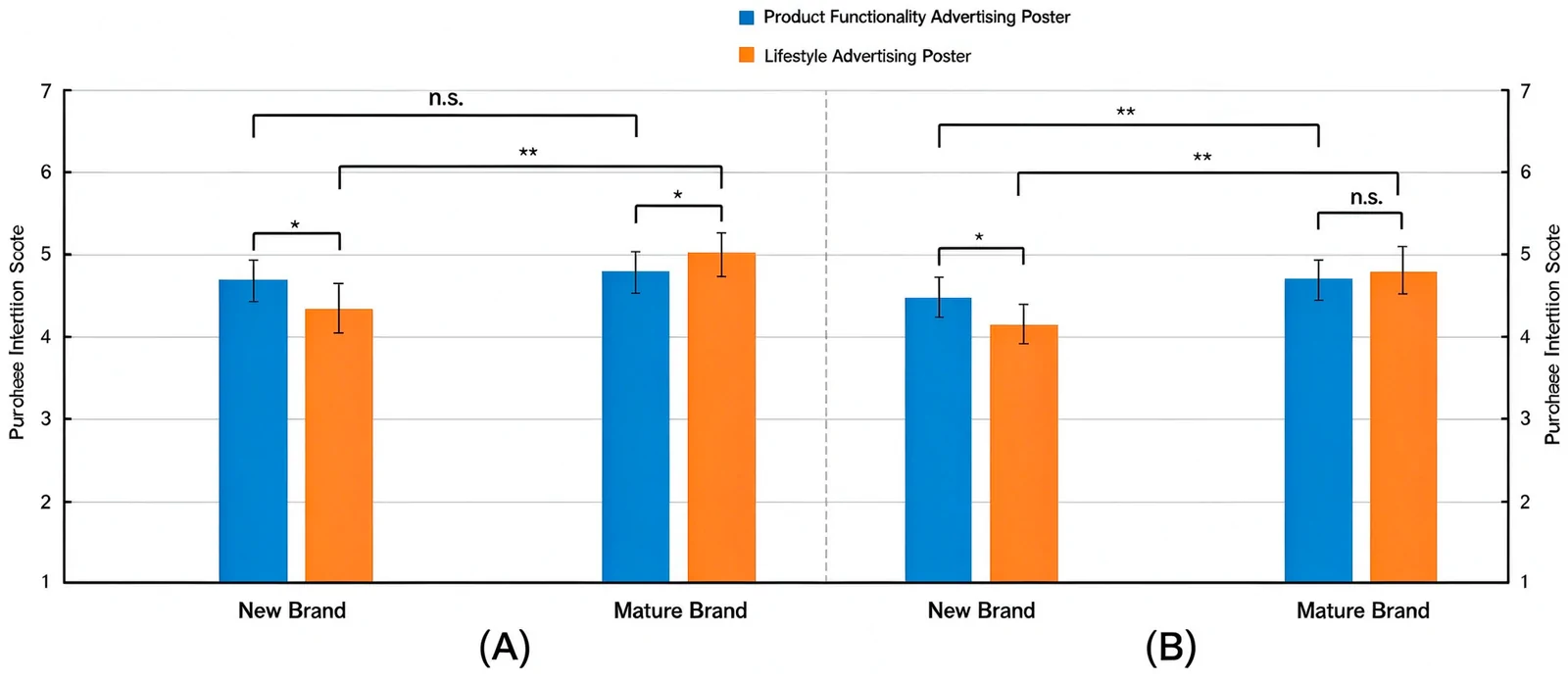
Purchase interest (**A**) and purchase possibility (**B**) under various experimental treatments of study 3a. * represents *p* < 0.05, ** represents *p* < 0.01, and n.s. represents non-significant.

Additionally, in the lifestyle slogan condition, the purchase interest of mature brands (*M* = 5.04, *SD* = 0.87) was significantly higher than that of new brands (*M* = 4.37, *SD* = 0.96), *F*(1, 44) = 54.11, *p* < 0.001. In the functional slogan condition, there is no significant difference in purchase interest between the new brand and the mature brand, *F*(1, 44) = 3.67, *p* = 0.392, see Figure 7.

#### 9.2.2. Purchase Possibility

For purchase possibility, the results showed that the main effect of brand maturity was significant, *F*(1, 44) = 22.70, *p* < 0.001, η^2^ = 0.34. The main effect of advertising appeal was not significant, *F*(1, 44) = 0.59, *p* = 0.259, η^2^ = 0.03. The interaction between brand maturity and advertising slogan style was significant, *F*(1, 44) = 38.55, *p* < 0.001, η^2^ = 0.47.

Further simple effects analysis showed that, for the new brand, the purchase possibility elicited by the functional slogan (*M* = 4.49, *SD* = 0.86) was significantly higher than that elicited by the lifestyle slogan (*M* = 4.15, *SD* = 0.90), *F*(1, 44) = 8.50, *p* = 0.006. However, there was no significant difference in purchase probability of the mature brand between the functional slogan (*M* = 4.71, *SD* = 0.81) and the lifestyle slogan (*M* = 4.82, *SD* = 0.94), *F*(1, 44) = 1.10, *p* = 0.301.

In the functional slogan condition, the purchase probability of the mature brand (*M* = 4.71, *SD* = 0.81) was significantly higher than that of the new brand (*M* = 4.49, *SD* = 0.86), *F*(1, 44) = 5.59, *p* = 0.023. In the lifestyle slogan condition, the purchase possibility of mature brands (*M* = 4.82, *SD* = 0.94) was also significantly higher than that of new brands (*M* = 4.15, *SD* = 0.90), *F*(1, 44) = 41.68, *p* < 0.001 (see Figure 7).

Study 3a largely supported our hypotheses: functional slogans were more effective in increasing purchase intention for new brands, whereas lifestyle slogans were more effective for mature brands with higher brand awareness. These effects were stronger than those observed with fictitious brands in Study 2a. Following the design of Study 2b, Study 3b examines whether this pattern extends to posters combining images and text for real brands at different stages of maturity.

## 10. Study 3b

Study 3b mainly adopted the form of image–text advertising posters to explore the above effect on purchase intention. In addition, since real brand names were used, there may be differences in the degree of creativity in image–text advertising design; hence, Study 3b also measured the brand likability of real brands and the degree of creativity in advertising design. The hypothesis of Study 3b was the same as Study 2b.

### 10.1. Participants and Procedure

Based on G*Power 3.1 calculations, for the mixed-design repeated-measures ANOVA used in this study, a minimum total sample size of 54 participants was projected to achieve 95% statistical power (1 − β) at α = 0.05 with a medium effect size of *f* = 0.25. A total of 108 participants were recruited. Eight participants were excluded due to failure on the attention test, and 100 participants (46 males and 54 females) were retained in the subsequent analysis. The age range of the participants was 18–27 years (*M* = 20.68, *SD* = 2.08).

**Figure 8:**
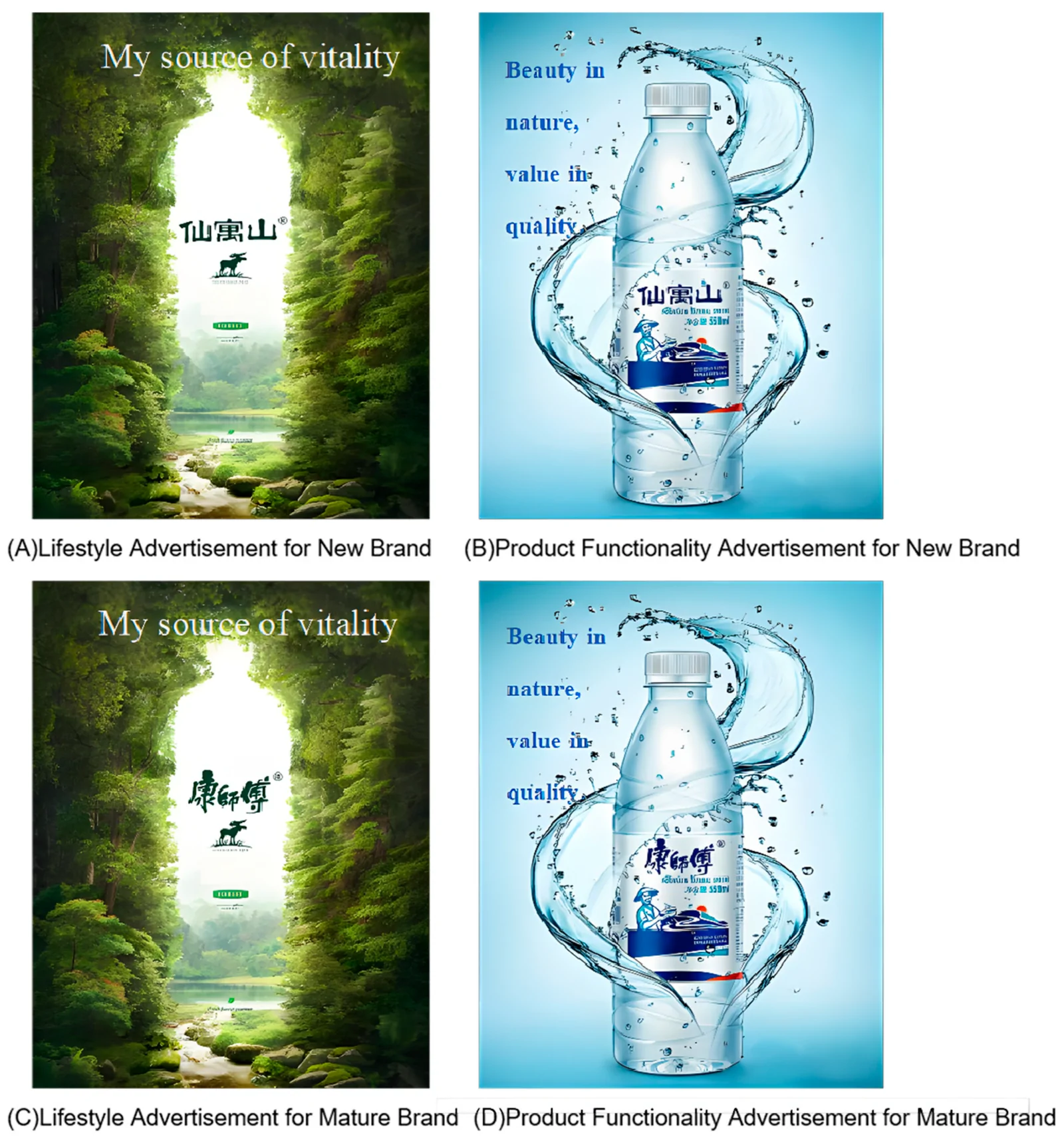
Examples of image–text advertising posters.

Study 3b used real brand names, and image–text advertising posters were rated as in Study 2b. Forty posters were selected, including 20 functional and 20 lifestyle posters. A paired-samples *t*-test showed that functional posters scored higher on product function attributes (*M* = 5.79, *SD* = 0.58) than lifestyle posters (*M* = 3.32, *SD* = 1.07), *t*(29) = 9.85, *p* < 0.001. Four sets of materials were then generated, each containing 20 posters. Examples are shown in Figure 8.

Study 3b adopted a 2 (brand maturity: new brand vs. mature brand; between-subjects) × 2 (advertising appeals: functional appeal vs. lifestyle appeal; within-subjects) mixed design.

The procedure of Study 3b was similar to Study 2b. Before rating purchase intention, participants evaluated brand likability and poster creativity on a 7-point scale (1 = very low; 7 = very high). In subsequent analyses, these variables were included as covariates to control for their potential effects on purchase intention.

### 10.2. Results and Discussion

#### 10.2.1. Purchase Interest

Brand likability and advertising creativity were controlled as covariates in Study 3b. For purchase interest, neither the main effect of advertising appeal nor brand maturity reached significance, for main effect of advertising appeal: *F*(1, 94) = 0.70, *p* = 0.403, η^2^ = 0.01, for main effect of brand maturity, *F*(1, 94) = 0.36, *p* = 0.548, η^2^ < 0.01, yet their interaction was significant, *F*(1, 94) = 32.31, *p* < 0.001, η^2^ = 0.26.

Simple effect analyses revealed functional posters generated greater purchase interest for new brands (*M* = 4.42, *SD* = 0.89) than lifestyle posters (*M* = 4.31, *SD* = 0.72), *F*(1, 94) = 4.28, *p* = 0.041. For mature brands, lifestyle posters yielded significantly higher purchase interest (*M* = 4.91, *SD* = 0.72) than functional posters (*M* = 4.64, *SD* = 0.77), *F*(1, 94) = 40.30, *p* < 0.001.

**Figure 9:**
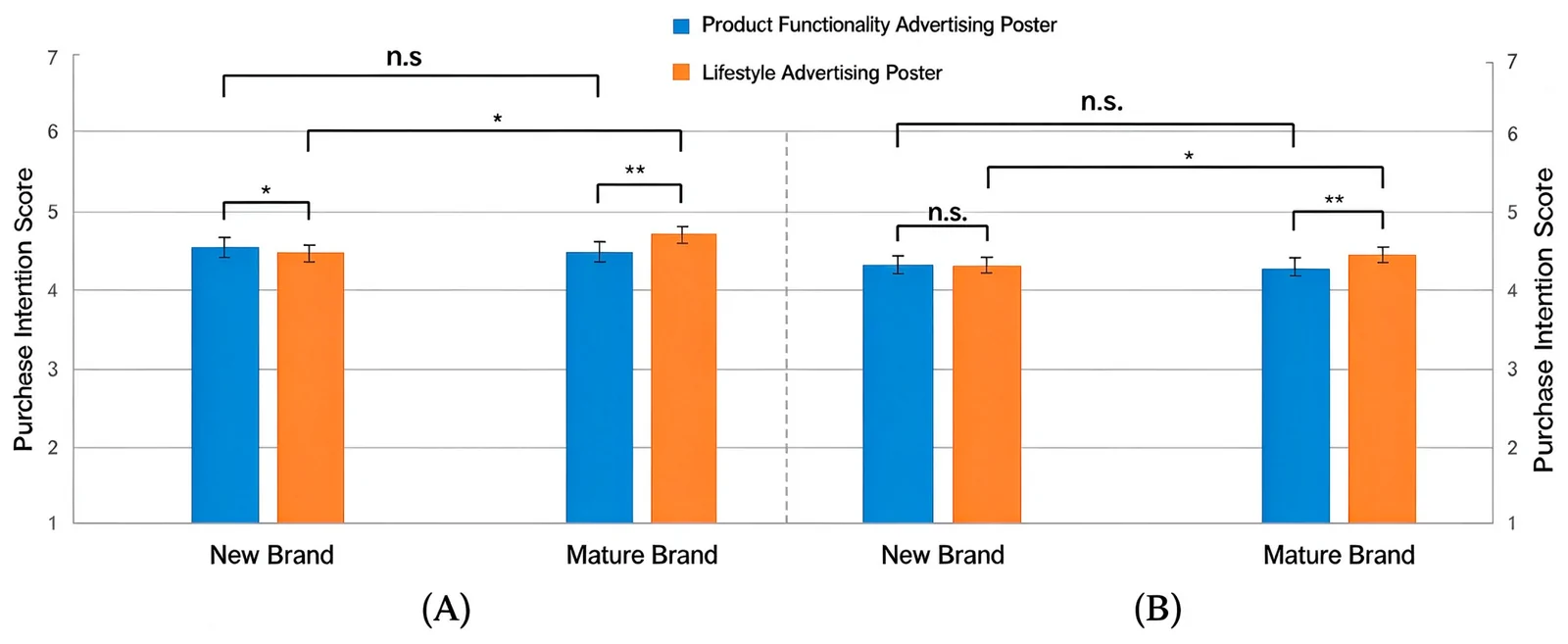
Purchase interest (**A**) and purchase possibility (**B**) under various experimental treatments of Study 3b. * represents *p* < 0.05, ** represents *p* < 0.01, and n.s. represents non-significant.

In the lifestyle advertising condition, the purchase interest of the mature brand (*M* = 4.91, *SD* = 0.72) was significantly higher than that of the new brand (*M* = 4.31, *SD* = 0.72), *F*(1, 94) = 5.70, *p* = 0.019. However, in the functional advertising condition, there was no significant difference in purchase interest between the new brand and the mature brand, *F*(1, 94) = 1.05, *p* = 0.309; see Figure 9.

#### 10.2.2. Purchase Possibility

For purchase possibility, after excluding the influence of brand likability and the degree of creativity in advertising design, we found the main effect of advertising appeal was not significant, *F*(1, 94) = 0.05, *p* = 0.824, η^2^ < 0.01. The main effect of brand maturity was not significant, *F*(1, 94) = 0.32, *p* = 0.570, η^2^ = 0.003. The interaction between advertising appeal and brand maturity was significant, *F*(1, 94) = 16.61, *p* < 0.001, η^2^ = 0.15.

Simple effects test indicated that lifestyle image–text posters generated significantly higher purchase possibility for mature brands (*M* = 4.83, *SD* = 0.74) than functional posters (*M* = 4.57, SD = 0.78), *F*(1, 94) = 26.86, *p* < 0.001. There was no significant difference in purchase possibility of a new brand between product functionality (*M* = 4.31, *SD* = 0.84) and lifestyle image–text advertising poster condition (*M* = 4.23, *SD* = 0.75), *F*(1, 94) = 0.73, *p* = 0.396.

In the lifestyle advertising condition, the purchase possibility of the mature brand (*M* = 4.83, *SD* = 0.74) was significantly higher than that of the new brand (*M* = 4.23, *SD* = 0.75), *F*(1, 94) = 4.08, *p* = 0.027. However, in the functional advertising condition, there was no significant difference in purchase possibility between new brand (*M* = 4.31, *SD* = 0.84) and mature brand (*M* = 4.57, *SD* = 0.78), *F*(1, 94) = 0.70, *p* = 0.404.

The results of Study 3b were largely consistent with those of Study 3a. Compared with fictitious brands, the effects of brand maturity and advertising appeal on purchase intention were more pronounced when real brands were used. Specifically, functional image–text advertisements were more effective for new brands, whereas lifestyle advertisements showed stronger effects for mature brands. Moreover, these effects remained significant after controlling for brand likability and advertising creativity, further supporting the robustness of the hypotheses.

## 11. Findings and Discussion

We introduce the concept of brand life cycle and examine how advertising appeal types influence purchase intention across different levels of brand maturity through six experiments. Study 1, using free association and forced-choice tasks, provides initial evidence of a matching effect: consumers focus more on functional appeal for new brands and show a stronger preference for lifestyle appeal for mature brands. This may be because consumers respond more to argumentative information when first encountering advertising, while their processing of rational information decreases with increasing familiarity (G. Li et al., 2016).

Study 2 further examined the proposed matching effect using fictitious brand names, with text-only advertisements in Study 2a and image–text advertisements in Study 2b. Because these fictitious brands do not exist in the marketplace, participants had no prior experience with them. This design helped minimize pre-existing differences in brand familiarity and brand evaluations across conditions, allowing us to examine the role of perceived brand maturity with fewer confounding influences. However, the use of fictitious brands also introduced an important limitation: participants may have had difficulty forming clear and credible perceptions of whether a hypothetical brand was new or mature.

In Study 2a, purchase intention was higher for mature brands and functional appeals, but the interaction between brand maturity and advertising appeal type was not significant. The absence of the predicted matching effect may be partly attributable to the limited information provided by the text-only advertisements and the difficulty of establishing clear perceptions of brand maturity. Without established brand associations, participants may have been less able to distinguish between hypothetical new and mature brands, weakening the predicted interaction between brand maturity and advertising appeal.

Study 2b provided partial support for the proposed matching effect. For mature brands, lifestyle appeals elicited higher purchase intention than functional appeals, consistent with research suggesting that emotional and experiential advertising cues can enhance consumers’ favorable responses to established brands (Machleit et al., 1993; Teixeira et al., 2012). However, advertising appeal type did not significantly affect purchase intention for new brands. This asymmetry may again reflect the limitations of fictitious brands. Although fictitious brands reduce differences in prior familiarity and existing brand evaluations, their names lack the pre-existing associations that consumers can draw on when forming judgments about real brands (de Lencastre et al., 2023; Grappi et al., 2024). The richer visual information provided by image–text advertisements may have partially compensated for this limitation, which may explain the emergence of the predicted effect among mature brands in Study 2b. Nevertheless, the complete matching pattern was not observed.

To address this limitation, Study 3 employed real brands, which carry consumers’ pre-existing knowledge of brand history and market position, enabling strong and reliable perceptions of brand maturity. Notably, Study 3a used text-only advertising stimuli, yet the matching pattern emerged: functional appeals increased purchase intention for new brands, whereas lifestyle appeals were more effective for mature brands, particularly for purchase interest. These effects remained significant after controlling for brand likability and advertisement creativity. The results of Study 3b were largely consistent with those of Study 3a. Although real brands naturally vary in brand familiarity, the consistent pattern observed in Study 3, together with the evidence from Study 2, suggests that the matching effect cannot be explained solely by brand familiarity. This discrepancy suggests that the matching effect is more robust for purchase interest, while purchase possibility is susceptible to additional confounding factors such as personal traits, price considerations, and situational constraints that mediate the translation from purchase intention to behavioral tendency (X. Wang et al., 2025).

We speculate that this effect may stem from consumers’ prior knowledge and the information-processing motivation evoked by such knowledge (Maclnnis et al., 1991; Romaniuk & Nicholls, 2006; Yi, 1993). Existing research has demonstrated that the effectiveness of advertising appeals is shaped by individuals’ prior knowledge (Albarracín & Shavitt, 2018; Lin & Lin, 2022). Prior knowledge constitutes a core component of the Elaboration Likelihood Model (Petty & Cacioppo, 1986). According to the fundamental tenets of the ELM, information-processing routes exert influences on consumers’ preference patterns. Specifically, the ELM posits that recipients of persuasive messages may adopt either the central or peripheral route, depending on their motivation and cognitive ability. When recipients possess high motivation (e.g., risk perceptions, high personal relevance of information) and high ability (e.g., absence of distraction, abundant prior knowledge) to think about message content, they tend to scrutinize issue-relevant arguments and process information via the central route. By contrast, in the absence or deficiency of motivation or cognitive ability, individuals follow the peripheral route (El Hedhli & Zourrig, 2023). Furthermore, prior knowledge serves as a critical determinant of people’s ability and motivation to process incoming information: the more relevant knowledge individuals hold, the more likely they are to engage in thorough processing of theme-related information (Sakaki et al., 2024; Wood et al., 1985).

For new brands, consumers typically have limited prior knowledge and few established associations with the brand or its products. Limited knowledge may increase consumers’ motivation to process information about unfamiliar products and marketing stimuli (Grunert, 1996; Hüttl-Maack et al., 2024). At the same time, new brands have not yet accumulated substantial reputational capital, which may increase uncertainty about product quality and functionality and, consequently, perceived choice risk. Under these conditions, advertising can serve as an important source of diagnostic product information. Consumers may therefore be more motivated to examine concrete information about product attributes and functional benefits, making functional appeals particularly relevant to their evaluations of new brands.

The situation may differ for mature brands. Through repeated advertising exposure, product experience, and word-of-mouth communication, consumers have accumulated more knowledge and established associations related to these brands. This existing knowledge may reduce the need to process additional functional information. Consistent with this possibility, prior research has shown that greater familiarity with an object can reduce interest and induce boredom (Pechmann & Stewart, 1988; Schmidt & Eisend, 2015). Similar patterns have been observed in consumer behavior: McAlister and Pessemier (1982) found that consumers may switch among familiar brands to alleviate boredom. Thus, repeated functional appeals may provide relatively little new information for consumers who are already familiar with mature brands. Under these conditions, consumers may respond more favorably to lifestyle appeals, which emphasize emotional experiences, symbolic meanings, and connections with consumers’ lifestyles.

Overall, the findings are consistent with an ELM-based explanation in which brand maturity is associated with differences in consumers’ prior knowledge, perceived uncertainty, and motivation to process advertising information. For new brands, limited knowledge and greater uncertainty may increase the value of concrete, diagnostic information, making functional appeals more effective. For mature brands, accumulated knowledge may reduce the need for further processing of familiar functional information, increasing the relative appeal of lifestyle appeals. Thus, the matching effect may reflect differences in the type of information consumers seek and process as their knowledge and experience with a brand increase, rather than a simple correspondence between functional appeals and the central route or between lifestyle appeals and the peripheral route. However, the ELM provides a framework for interpreting the pattern, but the proposed explanation remains theoretically inferred rather than empirically established.

An information-processing perspective suggests several possible explanations that require direct testing. The ELM links message elaboration to recipients’ motivation and ability to evaluate relevant information (Petty & Cacioppo, 1986). In this context, uncertainty may influence consumers’ motivation to learn about an offering. If consumers are uncertain about a new brand, concrete functional information may clarify its attributes and expected benefits. The resulting reduction in uncertainty could help explain more favorable purchase-intention responses. An appeal-induced reduction in uncertainty would be a candidate mediator. Identifying the processes that generate these differences requires direct measurement and experimental testing.

## 12. Contributions, Implications and Future Research Directions

### 12.1. Theoretical Contributions

First, grounded in the brand life cycle perspective, this study explores the fit between distinct types of advertising appeals and brands at different maturity stages, while examining whether this effect remains consistent across text-only and image–text combined advertisements. The findings advance our understanding of consumer purchase decision-making and enrich the literature and theoretical accounts regarding how advertising appeal styles shape consumer responses. Second, this research lies at the intersection of advertising and consumer psychology. As such, the systematic investigation of this phenomenon offers new theoretical perspectives and research avenues for future interdisciplinary work bridging advertising and consumer psychology, carrying important methodological implications.

### 12.2. Practical Contributions

New brands and mature brands represent two distinct brand life cycle stages with unique market characteristics. It is critical for marketers to select appropriate advertising appeal types to attract consumers and boost purchase intentions in a targeted manner. The findings of this study can guide firms to develop more effective advertising strategies, help them better capture market trends and consumer needs, and use advertising as a vital medium to communicate brand value and product information, thereby enhancing market competitiveness. Accordingly, this research carries substantial practical significance.

### 12.3. Implications

Based on the findings, this study offers several recommendations for marketers in formulating advertising strategies. For new brand advertisements, marketers should first emphasize product function, focusing on specific parameters such as the functions, quality, and performance. They should demonstrate how the product meets consumers’ needs in a clear and concrete manner to highlight its unique functions and advantages, thereby enhancing purchase intention and helping the brand stand out in a competitive market. Additionally, since consumers may have little knowledge of new brands, advertising for new brands should also include educational content regarding product usage and performance, which also helps build consumers’ cognition and trust in the product.

For mature brands, since consumers are already familiar with their specific parameters, marketers should focus on brand image building when formulating advertising strategies. Specifically, mature brands should convey a lifestyle and values that resonate with consumers through advertisements, which helps deepen the emotional connection between consumers and the brand. Additionally, mature brands need to leverage lifestyle appeals and convey lifestyles that resonate with consumers through new creative, emotional, and conceptual expression methods. This injects fresh elements into advertisements, thereby avoiding repetition and monotony in advertising content.

### 12.4. Limitations and Future Research

This study has certain limitations. First, the product categories used as experimental materials were concentrated on fast-moving consumer goods, and the product variety was limited. In addition, the participants recruited in our experiments were relatively young, and the sample sizes were modest. Fast-moving consumer goods fit better with the fast-paced lifestyles of young consumers. Accordingly, young people’s advertising responses and product evaluations for them may differ from those of older adults and consumers purchasing durable goods, which may limit the generalizability of our results. Future research could recruit participants across a wider age spectrum. J. Wang (2018) argued that there is also a matching effect between product type and advertising appeal style on purchase intention and product attitude. Therefore, incorporating product type into consideration is also a direction for future research.

Second, this study measured only self-reported purchase intention as the dependent variable. The lack of behavioral indicators leads to certain limitations in the ecological validity of the research. Future research could adopt behavioral measures such as real choice tasks or actual sales data to validate and extend these results.

Third, brand maturity and brand familiarity share conceptual similarities. Although maturity was operationalized through brand history and perceived developmental status, the real-brand selection criteria also incorporated public awareness, which may covary with consumers’ familiarity and brand knowledge. Product life cycle stage moderates the effect of brand familiarity on consumer attitudes (Ladeira et al., 2022). Future research could independently manipulate brand history cues and prior exposure, while separately measuring perceived maturity, familiarity, and brand knowledge. Such designs would help assess whether the matching pattern depends on developmental stage information, accumulated consumer experience, or their combination.

In addition, the proposed psychological mechanism was not directly tested. This study did not measure or formally test the mediating roles of perceived uncertainty, elaboration, information diagnosticity, or brand knowledge. It cannot conclusively verify the causal pathway through which it emerges. Future research could measure these candidate processes and test indirect effects, which could distinguish among competing explanations.

Finally, this study mainly focused on examining how advertising appeal types influence the purchase intention of brands at different maturity stages. However, previous studies have found that factors such as motivation (Maclnnis et al., 1991), prior knowledge (Albarracín & Shavitt, 2018), and identity (Y. Li et al., 2024) all affect consumer attitudes and behaviors. Thus, future research can further explore the potential psychological mechanisms underlying this effect.

## Data Availability

The original contributions presented in this study are included in the article. Further inquiries can be directed to the corresponding authors.
